# Sex-Specific Effects on Spatial Learning and Memory, and Sex-Independent Effects on Blood Pressure of a <3.3 Mbp Rat Chromosome 2 QTL Region in Dahl Salt-Sensitive Rats

**DOI:** 10.1371/journal.pone.0067673

**Published:** 2013-07-04

**Authors:** Victoria L. Herrera, Khristine A. Pasion, Glaiza A. Tan, Ann Marie Moran, Nelson Ruiz-Opazo

**Affiliations:** Section of Cardiovascular Medicine, Department of Medicine, Boston University School of Medicine, Boston, Massachusetts, United States of America; The University of Tennessee Health Science Center, United States of America

## Abstract

Epidemiological studies have consistently found that hypertension is associated with poor cognitive performance. We hypothesize that a putative causal mechanism underlying this association is due to genetic loci affecting both blood pressure and cognition. Consistent with this notion, we reported several blood pressure (BP) quantitative trait loci (QTLs) that co-localized with navigational performance (Nav)-QTLs influencing spatial learning and memory in Dahl rats. The present study investigates a chromosome 2 region harboring *BP-f4* and *Nav-8* QTLs. We developed two congenic strains, S.R2A and S.R2B introgressing Dahl R-chromosome 2 segments into Dahl S chromosome 2 region spanning *BP-f4* and *Nav-8* QTLs. Radiotelemetric blood pressure analysis identified only S.R2A congenic rats with lower systolic blood pressure (females: −26.0 mmHg, *P* = 0.003; males: −30.9 mmHg, *P*<1×10^−5^), diastolic blood pressure (females: −21.2 mmHg, *P* = 0.01; males: −25.7 mmHg, *P*<1×10^−5^), and mean arterial pressure (females: −23.9 mmHg, *P* = 0.004; males: −28.0 mmHg, *P*<1×10^−5^) compared with corresponding Dahl S controls, confirming the presence of *BP-f4* QTL on rat chromosome 2. The S.R2B congenic segment did not affect blood pressure. Testing of S.R2A, S.R2B, and Dahl S male rats in the Morris water maze (MWM) task revealed significantly decreased spatial navigation performance in S.R2A male congenic rats when compared with Dahl S male controls (P<0.05). The S.R2B congenic segment did not affect performance of the MWM task in males. The S.R2A female rats did not differ in spatial navigation when compared with Dahl S female controls, indicating that the *Nav-8* effect on spatial navigation is male-specific. Our results suggest the existence of a single QTL on chromosome 2 176.6–179.9 Mbp region which affects blood pressure in both males and females and cognition solely in males.

## Introduction

Essential hypertension is a leading risk factor for myocardial infarction and stroke [1], [2], and epidemiologic studies have shown that cardio- and cerebrovascular diseases are related to cognitive decline [3]–[6]. Moreover, high systolic blood pressure (SBP) occurring in midlife increases the risk of developing clinical dementia at older ages [7], [8]. These findings have been extended even to the normotensive range, in which increasing systolic blood pressure in the high-normal blood pressure range is associated with poor cognitive performance [9]. The relationship between high blood pressure and dementia was further demonstrated by showing that antihypertensive treatment reduces the incidence of subsequent dementia [10], [11].

Hypertension and dementia are conditions that exhibit highly complex inheritance involving environmental, genetic, and epigenetic mechanisms. These disorders are further compounded by phenotype variation due to its relatively late onset, variable disease course, sex-specific differences, and emerging impact of gestational environmental factors. Thus, animal models offer the ability to eliminate major confounds from diet and developmental programming and conduct controlled genetic experiments to localize quantitative trait loci (QTLs) on their genomes affecting blood pressure [12] and cognition [13]. We hypothesize that the reported association between high blood pressure and cognitive performance can be explained in part by the pleiotropic effects of loci affecting both blood pressure and cognition. Thus, we initially tested this notion by performing a genome-wide scan for QTLs influencing blood pressure and spatial navigational performance in F2-intercross rat populations derived from the same Dahl salt-resistant (Dahl R/jr^HS^) and Dahl salt-sensitive (Dahl S/jr^HS^) inbred rat lines. We detected several blood pressures (BP)-QTLs [12] that co-localized with spatial navigation (Nav)-QTLs affecting spatial learning and memory performance [13]. This raises the possibility that genes underlying these overlapping QTLs might represent pleiotropic effects of single gene variants. The present study was undertaken to initially test this hypothesis by 1) confirming the presence of one BP-QTL and one Nav-QTL in a chromosome 2 region harboring *BP-f4* [12] and *Nav-8* [13] QTLs, 2) delimiting more precisely the chromosomal region (s) harboring these two QTLs, and 3) determining whether the *BP-f4* and *Nav-8* QTLs would remain within that same congenic segment, a finding that would support the hypothesis that a single gene variant might affects both traits.

## Results

**Figure 1:**
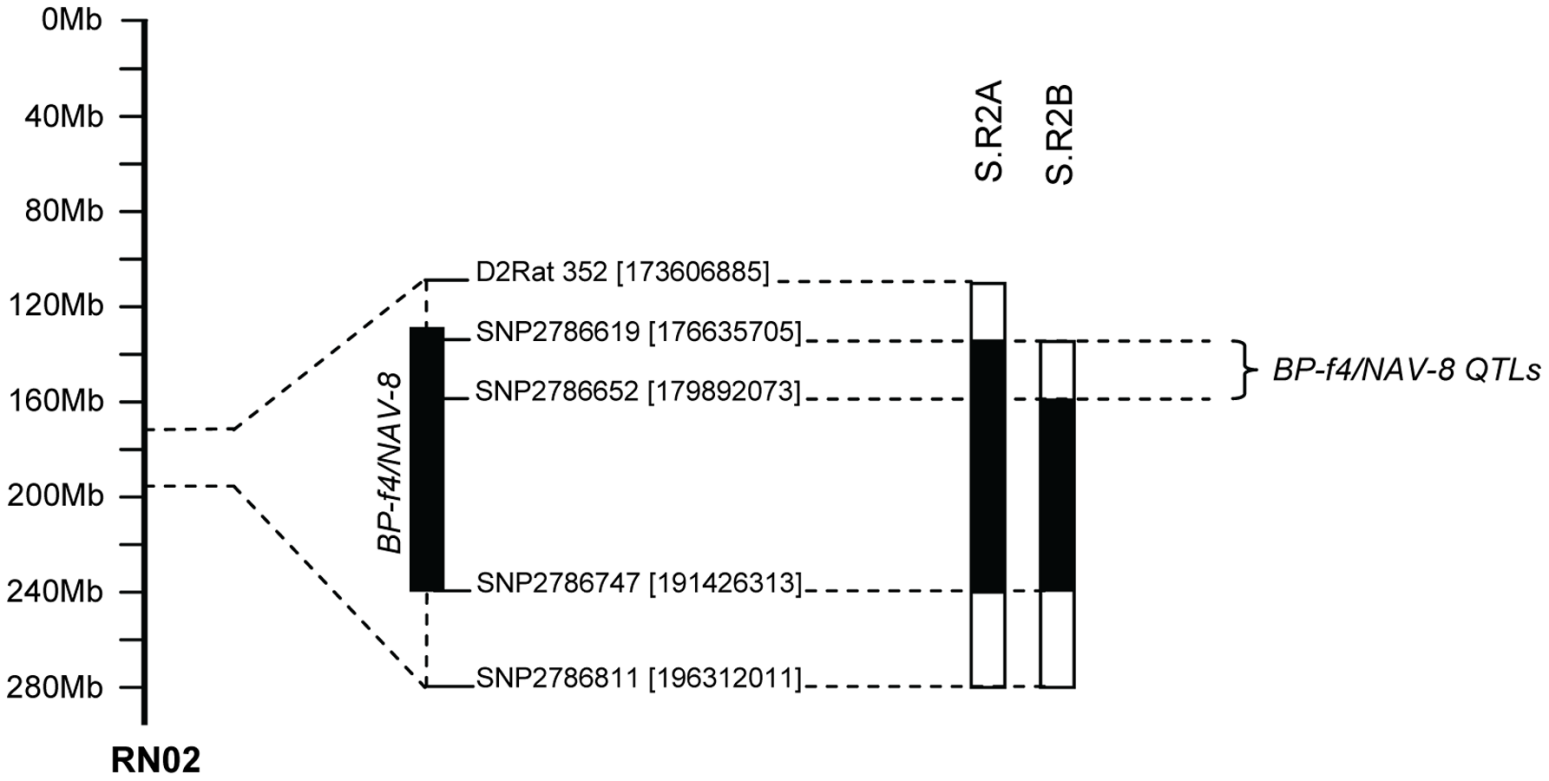
Congenic analysis of *BP-f4/Nav-8* QTL region on chromosome 2. On the left of the figure is shown the relevant region of the physical map of rat chromosome 2. Values in parenthesis next to the marker names denote physical locations in base pairs. The mapped *BP-f4/Nav-8* QTL region of approximately 3.26 Mbp is noted (to the right). Congenic strains are shown as solid bars (representing the Dahl R introgressed fragments) flanked by open bars (representing the putative regions of recombination).

Our two previous linkage studies performed on two F2 (Dahl S×R)-intercross rat populations, one cohort phenotyped for blood pressure [12] and another population characterized for navigational performance [13], delineated the potential existence of two closely linked QTLs on chromosome 2, one affecting blood pressure (*BP-f4*) [12] and the other affecting navigational performance (*Nav-8*) [13] (Table 1). To confirm the existence of these two QTLs in this region, we transferred two Dahl R chromosomal segments spanning the *BP-f4/Nav-8* QTLs onto the Dahl S genetic background (Figure 1). We successfully implemented a “speed congenic” strategy towards the development of highly inbred S.R2A and S.R2B (Figure 1) congenic lines. We performed back-crosses up to back-cross 6 and established homozygous congenic lines for blood pressure measurements and assessment of navigational performance in the Morris water maze (MWM) task. For back-cross 6, the S.R2A congenic line was greater than 99.70% of the Dahl S genetic background and the S.R2B congenic line was greater than 99.77% of the Dahl S genetic background.

**Table 1:** Overlapping blood pressure and spatial navigation QTLs detected in F2 (Dahl S×R) intercross rats.

BP-QTLs [12]		Nav-QTLs [13]	
*QTL*	*Chr-location*	*QTL*	*Chr-location*
*BP-m2*	Chr1–208.8 Mbp	*Nav-3*	Chr1–197.7 Mbp
*BP-f4*	Chr2–181.7 Mbp	*Nav-8*	Chr2–176.7 Mbp
*BP-m5*	Chr11–48.0 Mbp	*Nav-7*	Chr11–48.0 Mbp
*BP-m3*	Chr20–35.7 Mbp	*Nav-12*	Chr20–32.6 Mbp

QTL, quantitative trait locus; BP, blood pressure; Nav, spatial navigation; Chr, chromosome.

Blood pressure analysis of the congenic lines harboring the chromosome 2 region spanning putative *BP-f4* and *Nav-8* QTLs (Figure 1) substantiated the existence of *BP-f4* QTL in this region as demonstrated by the significantly lower systolic, diastolic, and mean blood pressures exhibited by SR.2A congenic rats compared with Dahl S controls (Table 2). Introgression of the S.R2A congenic segment onto the Dahl S genetic background lowered systolic blood pressure by 26.0 mmHg (*P* = 0.003, Table 2), diastolic blood pressure by 21.2 mmHg (*P* = 0.01, Table 2), and mean arterial pressure by 23.9 mmHg (*P* = 0.004, Table 2) in females. Blood pressure in S.R2B congenic female rats did not differ from Dahl S female controls, demonstrating the absence of genes affecting blood pressure in this chromosomal region, thus delimiting the chromosomal region to less than 3.3 Mbp (176.6–179.9 Mbp, containing 36 annotated genes, Table 3) that harbors the gene underlying *BP-f4*. Similarly, S.R2A congenic males showed significantly lower systolic blood pressure (−30.9 mmHg, *P*<1×10^−5^, Table 2), diastolic blood pressure (−25.7 mmHg, *P*<1×10^−5^, Table 2), and mean arterial pressure (−28.0 mmHg, *P*<1×10^−5^, Table 2) when compared with male controls. Interestingly, we detected significant differences in pulse pressure between S.R2A and Dahl S control rats (*P*<0.05, Table 2), suggesting that the gene underlying *BP-f4* QTL affects arterial stiffness as well.

**Table 2:** Effects of female and male rat chromosome 2 congenic strains on blood pressure.

Strain	n		Δ	*P*
**Females**				
***SBP***				
Dahl S	9	194.4±6.2		
S.R2A	6	168.4±2.0	26.0	**0.003**
S.R2B	10	187.0±3.0	7.4	0.227
***DBP***				
Dahl S	9	139.7±5.8		
S.R2A	6	118.5±2.2	21.2	**0.010**
S.R2B	10	135.2±2.8	4.5	0.427
***MAP***				
Dahl S	9	165.9±5.9		
S.R2A	6	142.0±2.1	23.9	**0.004**
S.R2B	10	159.6±2.8	6.3	0.280
***PP***				
Dahl S	9	54.74±0.8		
S.R2A	6	50.00±1.0	4.74	**0.043**
S.R2B	10	51.84±1.4	2.90	0.147
**Males**				
***SBP***				
Dahl S	8	211.7±2.4		
S.R2A	6	180.8±3.7	30.9	**<1**×**10** ^−**5**^
***DBP***				
Dahl S	8	154.7±2.0		
S.R2A	6	129.0±2.9	25.7	**<1**×**10** ^−**5**^
***MAP***				
Dahl S	8	181.7±2.2		
S.R2A	6	153.7±3.1	28.0	**<1**×**10** ^−**5**^
***PP***				
Dahl S	8	57.1±0.4		
S.R2A	6	51.8±2.3	5.3	**0.024**

Values are means ± standard error of the means; n, number of animals; Δ, difference between Dahl S and congenic values; SBP, systolic blood pressure in mmHg; DBP, diastolic blood pressure in mmHg; MAP, mean arterial pressure in mmHg; PP, pulse pressure; *P* in females, one-way ANOVA followed by Holm-Sidak’s test for multiple comparisons; *P* in males, t-test.

**Table 3:** Annotated genes in rat chromosome 2 176.6–179.9 Mbp region.

*Symbol*	*Location*	*Description*
*LOC100360914*	176.67 Mbp	F-box and WD-40 domain protein 7, archipelago homolog (Drosophila)-like
***DEspR (Dear)***	**176.74 Mbp**	**Dual endothelin 1, vascular endothelial growth factor signal peptide receptor (Dual endothelin 1, angiotensin II receptor)**
*Pmf31*	176.76 Mbp	PMF32 protein
*LOC100360966*	176.83 Mbp	Hypothetical LOC100360966
*RGD1565157*	177.25 Mbp	Similar to testis-specific farnesyl pyrophosphate synthase
*RGD1565007*	177.64 Mbp	Similar to RIKEN cDNA 4632419K20
*LOC100361062*	177.81 Mbp	Hypothetical LOC100361062
*LOC295168*	177.85 Mbp	Hypothetical LOC295168
*LOC499648*	177.92 Mbp	Similar to cleavage and polyadenylation specificity factor 3
*LOC100361162*	177.93 Mbp	Hypothetical protein LOC100361162
*LOC365835*	177.96 Mbp	Similar to epidermis-specific serine protease-like
*Sh3d19*	178.04 Mbp	SH3 domain containing 19
*Rps3a*	178.10 Mbp	40S ribosomal protein S3a
*Lrba*	178.26 Mbp	LPS-responsive vesicle trafficking, beach and anchor containing
*Mab2112*	178.53 Mbp	Mab-21-like 2 (C. elegans)
*Dclk2*	178.80 Mbp	Doublecortin-like kinase 2
*Cd1d1*	179.02 Mbp	CD1d1 molecule
*LOC365837*	179.05 Mbp	Similar to Multisynthetase complex auxiliary component p43
*RGD1564674*	179.06 Mbp	Similar to high mobility group protein
*LOC100361304*	179.08 Mbp	Hypothetical LOC100361304
*Kirrel*	179.17 Mbp	Kin of IRRE like (Drosophila)
*Fcrls*	179.30 Mbp	Fc receptor-like S, scavenger receptor
*LOC365839*	179.32 Mbp	Similar to elongation protein 4 homolog
*Cd5l*	179.39 Mbp	Cd5 molecule-like
*Fcrl1*	179.42 Mbp	Fc receptor-like 1
*Fcrl3*	179.48 Mbp	Fc receptor-like 3
*Etv3*	179.57 Mbp	Ets variant 3
*Etv3l*	179.60 Mbp	Ets variant 3-like
*LOC100361532*	179.62 Mbp	Hypothetical LOC100361532
*RGD1563314*	179.63 Mbp	Similar to phosphoglycerate mutase (EC 5.4.2.1) B chain-rat
*LOC680772*	179.64 Mbp	Similar to heterogeneous nuclear ribonucleoprotein F
*Arhgef11*	179.67 Mbp	Rho guanine nucleotide exchange factor (GEF) 11
*RGD1309453*	179.80 Mbp	Similar to hypothetical protein FLJ32884
*Pear1*	179.81 Mbp	Platelet endothelial aggregation receptor 1
*Ntrk1*	179.84 Mbp	Neurotrophic tyrosine kinase, receptor, type 1
*Insrr*	179.86 Mbp	Insulin receptor-related receptor

Gene list and gene locations on rat chromosome 2 176.6–179.9 Mbp region as per NCBI Rat Genome Assembly v3.4.

Since we detected the *Nav-8* QTL in an F2 (Dahl S×R)-intercross male population [13], we evaluated the effect of transferring S.R2A and S.R2B congenic segments onto the Dahl S genetic background on spatial learning and memory by testing S.R2A, S.R2B, and Dahl S control male rats in the Morris water maze (MWM) task to evaluate visiospatial cognition [14].

**Figure 2:**
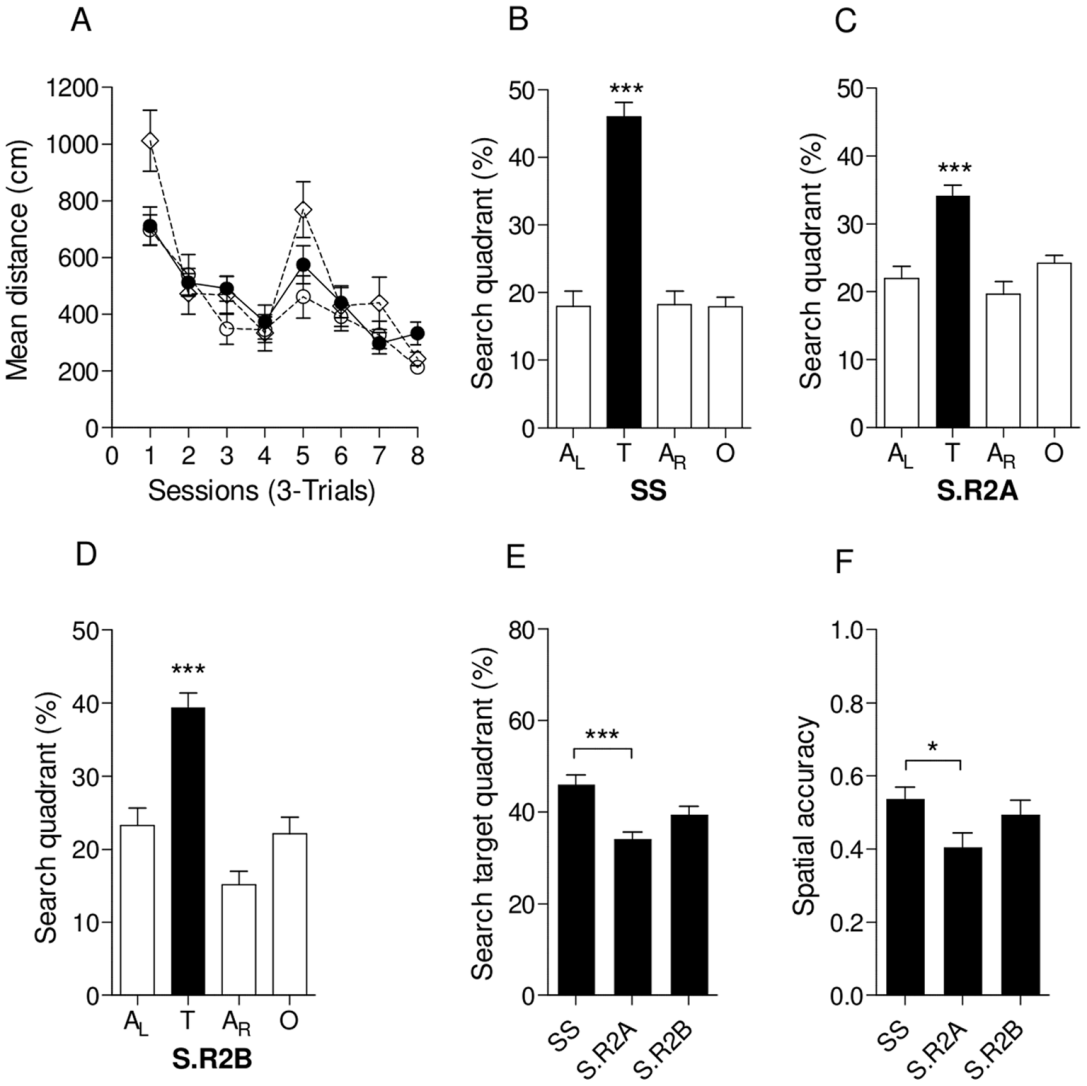
Testing of spatial learning and memory in Dhal S, S.R2A and S.R2B congenic male rats. (A) Acquisition performance measured as mean distance in cm during the 24 trials (•, Dahl S; ◊, S.R2A; ○, S.R2B). Percentage distance traveled in quadrants (B, C, D, E) and spatial accuracy performance (F) during the probe trial after completion of Morris water maze training. Quadrants are: target (T), opposite (O), adjacent right (A_R_) and adjacent left (A_L_). ^*^ *P*<0.05, ^***^ *P*<0.001. Data represent means ± s.e.m. (one-way ANOVA followed by Holm-Sidak’s test for quadrant occupancy in the probe trial of the Morris water maze task and for spatial accuracy performance).

Measurements of spatial learning in the MWM task revealed equivalent acquisition performance among all three groups (Figure 2A, *F*_2,336_ = 1.39, *P*>0.2). In the probe trial for spatial memory, all three groups exhibited target selectivity, showing enhanced preference for the target quadrant over other quadrants (Figure 2B, Dahl S male rats: *P*<0.001; Figure 2C, S.R2A male rats: *P*<0.001; Figure 2D, S.R2B male rats: *P*<0.001). However, direct comparison of selectivity for the target quadrant in the probe trial showed better performance of Dahl S male controls compared with S.R2A male subjects (Figure 2E, *P*<0.001) and equivalent performance when compared with S.R2B male subjects (Figure 2E, *P*>0.3). Consistently, Dahl S male rats showed increased spatial accuracy performance when compared with S.R2A male subjects (Figure 2F, *P*<0.05), confirming their better search accuracy for the hidden platform.

**Figure 3:**
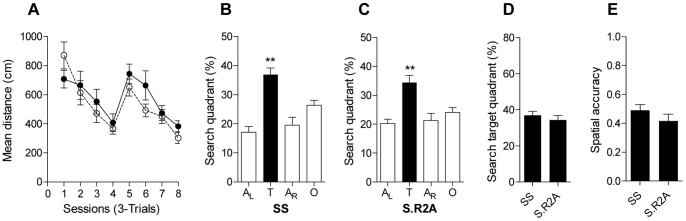
Testing of spatial learning and memory in Dhal S and S.R2A congenic female rats. (A) Acquisition performance measured as mean distance in cm during the 24 trials (•, Dahl S; ○, S.R2A). Percentage distance traveled in quadrants (B, C, D) and spatial accuracy performance (E) during the probe trial after completion of Morris water maze training. Quadrants are: target (T), opposite (O), adjacent right (A_R_) and adjacent left (A_L_). ^**^ *P*<0.01. Data represent means ± s.e.m. (one-way ANOVA followed by Holm-Sidak’s test for quadrant occupancy in the probe trial of the Morris water maze task and for spatial accuracy performance).

To investigate whether the decreased performance of S.R2A male subjects reflects a male-specific effect on spatial learning and memory or the effect of the congenic segment is independent of sex, we tested S.R2A female subjects and Dahl S female control rats in the MWM task. As shown in Figure 3, we observed no differences in acquisition (Figure 3A, *F*_1,216_ = 1.98, *P*>0.16) or in the probe trial performance between S.R2A female rats and Dahl S female controls. Both female groups demonstrated target selectivity (Figure 3B, Dahl S female rats: P<0.01; Figure 3C, S.R2A female rats: P<0.01) and equivalent performance in the probe trial, showing equivalent occupancy of target quadrant (Figure 3D) and spatial accuracy performance (Figure 3E).

## Discussion

Our earlier linkage studies showed evidence for two overlapping QTLs in the chromosome 2 176–182 Mbp region, one affecting blood pressure, *BP-f4* [12], and one affecting navigational performance, *Nav-8* [13], in F2 (Dahl S×R) rat intercrosses. The present study confirmed the existence of the *BP-f4* and *Nav-8* QTLs and co-localized these QTLs to a chromosome 2 region between 176.6–179.9 Mbp (Figure 1). Importantly, the introgression of the S.R2A Dahl R congenic fragment lowered blood pressure and worsened spatial learning and memory performance. These results agree with the expected Dahl R allele effects on blood pressure and spatial navigation performance detected in corresponding genome scans [12], [13]. Moreover, the gene underlying the *Nav-8* QTL affects navigational performance in a male-specific manner since both S.R2A congenic female rats and Dahl S female controls performed equivalently on MWM testing.

Epidemiologic studies have shown that hypertension is associated with cognitive decline [7]–[9]. However, in our congenic study in Dahl rats we found that introgression of a Dahl R congenic segment onto the Dahl S genetic background lowered blood pressure and worsened cognitive performance. This apparent discrepancy could be due to any of a number of characteristics related to gene function including a) specific gene variants affecting blood pressure and cognition in humans are likely to be different from gene variants affecting blood pressure and cognitive performance in animal models, b) directionality of specific gene variants effects on traits could differ by either increasing or decreasing trait values, c) the existence of different genetic modifiers (interacting loci) in rats and humans and d) the possibility of an age-dependent effect on these traits cannot be rule out since our studies were conducted in young subjects while epidemiologic investigations on hypertension and cognitive performance usually involve aging human populations.

Inspection of the chromosome 2 176.6–179.9 Mbp region spanning the *BP-f4* and *Nav-8* QTLs revealed the existence of 36 annotated genes (Table 3). One gene, *DEspR* (dual endothelin-1/vascular growth factor signal peptide receptor, formerly known as DEAR) at 176.74 Mbp, was shown to be a candidate gene underlying the *BP-f4* and *Nav-8* QTL-effects on blood pressure and spatial learning and memory. The remaining 35 annotated genes did not reveal obvious potential biological roles in blood pressure regulation or cognitive function. Several lines of evidence support that *DEspR* has pleiotropic effects on blood pressure and cognition: 1) the existence of one specific S44P/M74T DEspR variant that has been associated with salt-sensitive hypertension in Dahl rats [15], 2) DEspR haploinsufficiency in mice lowers blood pressure in female mice [16] and increases blood pressure in aging male mice [17], 3) a *DEspR-* T/CATAAAA-box promoter variant that decreases *DEspR* transcription has been associated with increased blood pressure in a male Sardinian population [17], and 4) DEspR haploinsufficiency in mice produces male-specific hippocampus-dependent cognitive deficits and improve hippocampus-dependent cognition in female mice [18]. Nevertheless, future fine mapping studies are necessary to verify *DEspR* as the gene underlying *BP-f4* and *Nav-8* QTLs.

Interestingly, a chromosome 4 scan for QTLs affecting general cognitive ability in humans detected several QTLs within the chromosome 4 120–180 Mbp region [19]. Notably, human *DEspR* maps to chromosome 4 153.26 Mbp and is a candidate gene underlying some of the observed chromosome 4 QTL effects on general cognitive ability [19]. The results of rat, mouse, and human studies support the hypothesis that *DEspR* has pleiotropic effects on both blood pressure and cognition.

Our study demonstrates the existence of either two closely linked QTLs or a single QTL on chromosome 2 176.6–179.9 Mbp region affecting blood pressure in both male and female Dahl S rats and influencing spatial learning and memory only in Dahl S male rats. Our results suggest the existence of a single QTL in the chromosome 2 176.6–179.9 Mbp region exhibiting pleiotropic effects on blood pressure and cognition with *DEspR* at chromosome 2 176.73 Mbp as a prime candidate gene underlying this QTL. The successful trapping of the chromosome 2 *BP-f4/Nav-8* QTL present in the S.R2A congenic line will form the basis to further fine map this QTL region to less than 0.1 Mbp via sub-strain construction and identify the specific gene variant accounting for this QTL. This would establish pleiotropy as an important genetic mechanism contributing to onset and development of different complex disorders and would explain in part the relationship between high blood pressure and poor cognitive performance observed in numerous epidemiological studies [3]–[11].

We detected three additional overlapping blood pressure and spatial navigation QTLs on chromosomes 1, 11, and 20, suggesting the existence of additional loci concomitantly affecting blood pressure and cognitive performance in Dahl rats. Thus, the elucidation of genetic mechanisms in complex traits would form an *a priori* basis for novel prevention and intervention strategies for complex disorders such as essential hypertension and cognitive impairment affecting the aging human population.

## Materials and Methods

### Ethics Statement

This study was performed in strict accordance with the recommendations in the Guide for the Care and Use of Laboratory Animals of the National Institutes of Health. The protocol was approved by the Committee on the Ethics of Animal Experiments of Boston University School of Medicine (Permit Number: AN-14966). All surgery was performed under sodium pentobarbital anesthesia, and every effort was made to minimize suffering.

### Strains

All rats utilized in this study were bred in-house. Inbred Dahl S/jrHsd and Dahl R/jrHsd rats were obtained from Harlan (Indianapolis, IN, USA). We transferred two Dahl R chromosomal segments spanning *BP-f4/Nav-8* onto the Dahl S genetic background. We implemented a “speed congenic” strategy [20], [21] to develop the two congenic lines. We first produced a (Dahl S×Dahl R) F1 progeny followed by generation of an F1×Dahl S backcross (BC1) population. We selected *BP-f4/Nav-8* “carriers” from 300 BC1 subjects by genotyping the BC1 male progeny with flanking markers of the chromosomal segments planned to be transferred. For S.R2A: sr heterozygous at SNP2786619 and SNP2786747, and ss homozygous at nearby flanking markers, i.e. D2Rat352 and SNP2786811. For S.R2B: sr heterozygous at SNP2786652 and SNP2786747, and ss homozygous at nearby flanking markers, i.e. SNP2786619 and SNP2786811. We produced 20 BC2 male subjects per congenic line and proceeded to screen subjects with 85 informative SNPs. One “best” male breeder per congenic line was chosen to continue with the inbreeding program. We performed back-crosses up to BC6 and established homozygous congenic lines for blood pressure measurements and Morris Water Maze performance. The SR2A congenic line was greater than 99.70% of Dahl S genetic background and the S.R2B congenic line was greater than 99.77% of Dahl S genetic background.

### Markers

We selected the following single nucleotide polymorphisms (SNPs) for congenic rat development from the rat genome data base (RGD): markers for S.R2A and S.R2B congenic fragments; SNP2786619, SNP2786747, D2Rat352, SNP2786811, SNP2786652. SNPs for implementation of “speed congenic” strategy, chr1: SNP2783361, SNP2783513, SNP2783573, SNP2783925, SNP2784073, SNP2784200, SNP2784723, SNP2784895, SNP2785046; chr2: SNP2785301, SNP2785499, SNP2785693, SNP2785860, SNP2786134, SNP2786276, SNP2786350, SNP2786619, SNP2786811, SNP2786979, SNP2787226; chr3: SNP2787599, SNP2787751, SNP2787947, SNP2788108, SNP2788217, SNP2788416; chr4: SNP2789191, SNP2789416, SNP2789717, SNP2789952, SNP2790223, chr5: SNP2790571, SNP2790733, SNP2790960, SNP2791234, SNP2791496, SNP2791711, SNP2791834; chr6: SNP2792065, SNP2792467, SNP2792754; chr7: SNP2793338, SNP2793565, SNP2793757, SNP2793904; chr8: SNP2794281, SNP2794450, SNP2794721, SNP2794865; chr9: SNP2795738, SNP2795947; chr10: SNP2796278, SNP2796474, SNP2796739, SNP2796966; chr11: SNP2797258, SNP2797443, SNP2797742; chr12: SNP2797924, SNP2798115; chr13: SNP2798475, SNP2798659, SNP2798785, SNP2798926; chr14: SNP2799254, SNP2799430, SNP2799825; chr15: SNP2800105, SNP2800195; chr16: SNP2800810, SNP2801108; chr17: SNP2801413, SNP2801584, SNP2801868, SNP2801948; chr18: SNP2802358, SNP2802507, SNP2802706; chr19: SNP2802997, SNP2803270; chr20: SNP2803540, SNP2803747; chrX: SNP2804065, SNP2804185, SNP2804233.

### Genotyping

DNA was extracted from tail biopsies using the QIAamp Tissue Kit (Qiagen, Hilden, Germany). SNP genotyping was carried out on a Life Technologies 7900 Real-Time PCR System (Foster City, CA, USA). All SNP assays (TaqMan assays) were procured from Life Technologies and were validated in our laboratory. Microsatellite markers were PCR genotyped and detected by 6% denaturing polyacrylamide gel electrophoresis.

### Blood Pressure Measurements

Animals were maintained on a Harlan 2018 rodent chow (Harlan Teklad, Madison WI) containing 0.23% NaCl from weaning until the high salt diet begun at 12 weeks of age. The food pellets and water were made available *ad libitum.* Blood pressure was measured essentially as described [12], [22] using intra-aortic abdominal radiotelemetric implants (DATASCIENCE) obtaining non-stressed blood pressure measurements taking the average over ten-seconds every 5 min for 24 h [12], [22]. Systolic, diastolic, and mean arterial pressures were obtained along with heart rate and activity. The protocol for the congenic and control rats was as follows: implant surgery at 10 weeks of age; after 12 days, baseline blood pressure levels were obtained. The high salt (8% NaCl) challenge was initiated at 12 weeks of age and maintained for four weeks for all rats as described [12], [22]. Blood pressure values used for phenotype comparison were the averages obtained for the last weekend of the salt loading from Friday–Monday with minimal entry to blood pressure room ascertaining non-stress blood pressure measurements.

### Morris Water Maze Testing

The MWM task was performed as described [13], [23]–[25] using a 1.5-m diameter, circular water maze (filled with water at 25°C±0.5), and a computer tracking system (Smart software program, Version 1.24, Panlab s.I., Barcelona, Spain). Distance was used to evaluate performance. Twelve swim trials were conducted per day (for 2 consecutive days). Animals were placed into the maze (at one of three randomized start positions located adjacent to the wall) and were allowed to traverse the maze in search of the escape platform. During each trial, a maximum swim time of 60 s was imposed. Between trials, a 35 s interval was imposed with the rat on the platform. At the end of the 24th trial, rats were removed to the home cage for 10 min, then the platform was removed (probe trial) and the rat was allowed to search for 1 min. Sixteen Dahl S males, 16 S.R2A males, 13 S.R2B males, 13 Dahl S females, and 16 S.R2A females were characterized in this MWM task for subsequent comparative analysis. Spatial accuracy was define as the ratio of % distance traveled in platform counter over the sum of % distance traveled over four counters localized at analogous positions on corresponding quadrants (Spatial accuracy = % distance Tcounter/% distance Tcounter+% distance A_L_counter+% distance A_R_counter+% distance Ocounter).

### Statistical Analyses

We performed a one-way analysis of variance (ANOVA) followed by all pairwise multiple comparisons using the Holm-Sidak test for blood pressures. We used a two-way repeated measures ANOVA followed by Holm-Sidak test for acquisition performance comparisons and a one-way ANOVA followed by Holm-Sidak test to analyze navigational performance on the probe trial.
